# The Dockstore: enabling modular, community-focused sharing of Docker-based genomics tools and workflows

**DOI:** 10.12688/f1000research.10137.1

**Published:** 2017-01-18

**Authors:** Brian D. O'Connor, Denis Yuen, Vincent Chung, Andrew G. Duncan, Xiang Kun Liu, Janice Patricia, Benedict Paten, Lincoln Stein, Vincent Ferretti

**Affiliations:** 1 UC Santa Cruz Genomics Institute, University of California, Santa Cruz, CA, USA; 2 Ontario Institute for Cancer Research, MaRS Centre, Toronto, Canada

**Keywords:** Docker, containers, genomics, bioinformatics, cloud, big data

## Abstract

As genomic datasets continue to grow, the feasibility of downloading data to a local organization and running analysis on a traditional compute environment is becoming increasingly problematic. Current large-scale projects, such as the ICGC PanCancer Analysis of Whole Genomes (PCAWG), the Data Platform for the U.S. Precision Medicine Initiative, and the NIH Big Data to Knowledge Center for Translational Genomics, are using cloud-based infrastructure to both host and perform analysis across large data sets. In PCAWG, over 5,800 whole human genomes were aligned and variant called across 14 cloud and HPC environments; the processed data was then made available on the cloud for further analysis and sharing. If run locally, an operation at this scale would have monopolized a typical academic data centre for many months, and would have presented major challenges for data storage and distribution. However, this scale is increasingly typical for genomics projects and necessitates a rethink of how analytical tools are packaged and moved to the data. For PCAWG, we embraced the use of highly portable Docker images for encapsulating and sharing complex alignment and variant calling workflows across highly variable environments. While successful, this endeavor revealed a limitation in Docker containers, namely the lack of a standardized way to describe and execute the tools encapsulated inside the container. As a result, we created the Dockstore ( https://dockstore.org), a project that brings together Docker images with standardized, machine-readable ways of describing and running the tools contained within. This service greatly improves the sharing and reuse of genomics tools and promotes interoperability with similar projects through emerging web service standards developed by the Global Alliance for Genomics and Health (GA4GH).

## Introduction

The Dockstore project has its roots in the large-scale ICGC PanCancer Analysis of Whole Genomes (PCAWG; https://dcc.icgc.org/pcawg) cancer genomics project, which necessitated the creation of highly portable and self-contained computational tools ^1^. PCAWG’s initial core goal was to consistently analyze approximately 2,800 cancer donors (~5,800 whole genomes), an effort that culminated in the re-alignment and somatic variant calling for these donors. This effort used considerable computational resources. At its peak, 14 cloud and HPC environments were utilized with over 16,000 cores in total, resulting in a cumulative dataset of nearly 1 Petabyte in size.

Our initial approach for PCAWG was to utilize cloud Application Program Interfaces (APIs) to build computational worker nodes from scratch, rather than use the Docker virtualization technology ^2^. In this approach, we used API calls to create virtual machines (VMs) and to install software on them using Linux Bash setup scripts and, later, Ansible playbooks ( https://www.ansible.com). We found that the use of cloud APIs and scripts to be a cumbersome and error prone way to move algorithms to the data. Over time dependencies and software versions would change, resulting in frequent failures of the setup scripts, or mysterious downstream analytical failures. Docker, a relatively new lightweight virtualization technology, mitigated these issues by providing a mechanism to encapsulate tools and their dependencies in a highly portable way ( https://www.docker.com). This meant PCAWG workflow authors could create and set up their environments within a Docker image, including tools, library dependencies, reference files, and so forth, and then copy that image from cloud to cloud for analysis of data in place. This allowed us to very quickly create cloud-based VMs, install Docker, pull the current version of the Docker-based workflows, and be ready to perform analysis within a few minutes, highly simplifying our deployment strategy. The consistent, portable execution environment provided within a Docker container meant we could avoid issues caused by differences between cloud environments. Furthermore, the inherent portability of Docker images allowed us to leverage a multitude of computational environments, including non-cloud environments that were previously inaccessible to the project.

Given our positive experience using Docker to distribute analytical tools, we began exploring a generalized method for other projects to leverage the same approach. Our creation, the Dockstore ( https://dockstore.org), generalizes the PCAWG approach in an easy-to-use web application that any tool developer or tool end user can utilize. The concept extends popular services used in Information Technology (IT) fields, in particular commercial sites, such as Quay.io ( https://quay.io) and DockerHub ( https://hub.docker.com), which provide hosted Docker registries where anyone can upload images containing tools or services.

Dockstore’s key innovation is its bridging of Docker image registries with a new, standardized approach to describing tools inside images. Up to this point, tools inside Docker images have had no standardized way to document how to call them, leading to the convention of using human-readable README files to describe tool invocation. This has made automation and integration among Docker images and execution systems cumbersome given the lack of machine-readable tool definitions. To solve this, we used the Common Workflow Language ( https://dx.doi.org/10.6084/m9.figshare.3115156.v2) or Workflow Definition Language (WDL; https://github.com/broadinstitute/wdl) tool definition syntaxes to define the commands available inside a Docker image, how to parameterize them, to describe their inputs/outputs and their resource requirements. Dockstore also supports linking multiple tools together using CWL or WDL workflows; these multi-image workflows can then be registered on the site and used as building blocks to create more complex systems. The result is that Dockstore-based tools and workflows can be programmatically addressed and executed, enabling a new level of modularity, automation and integration.

**Figure 1.**
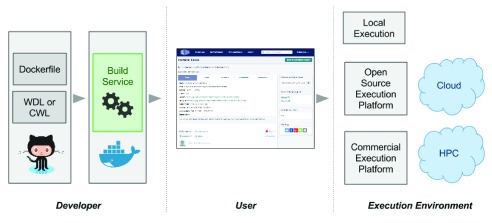
Use cases for Dockstore. Developers can use Dockstore to register Docker images built by, or uploaded to, Quay.io and DockerHub with CWL/WDL machine- and human-readable descriptors from GitHub or Bitbucket. Users can then query and find tools of interest, parameterize them, and run them at a small scale locally or at large scale on commercial or open source execution engines supporting Docker and CWL/WDL. Execution takes place on cloud or HPC environments supported by the execution engine of choice.

In addition to providing a mechanism to bring together Docker-based tools and their corresponding machine-readable descriptors, the Dockstore provides a compelling and useful web-based interface, an instance of which is hosted at https://dockstore.org. This allows it to serve two communities: developers who want to register and share their tools through Dockstore, and users wishing to find genomics tools packaged in Docker and ready to execute in their own systems ( Figure 1). The Dockstore web application provides a full host of capabilities for these two types of users, including registering new Docker images and descriptors, searching for tools others have registered, and assisting users in executing tools on any platform that supports Docker. The Dockstore also provides a command line interface for power users who want to script and automate their use of Dockstore.

Finally, Dockstore is supported by the Global Alliance for Genomics and Health (GA4GH) organization ^3^. The GA4GH’s mission is to accelerate progress in human health through establishing common frameworks for sharing genomics data and tools. The GA4GH Data Working Group focuses on data representation, storage, and analysis of genomic data. It provides an emerging standard web service API for accessing Docker-based tools and workflows ( https://github.com/ga4gh/tool-registry-schemas). This Tool Registry API is being developed as part of a larger effort by the GA4GH Containers and Workflows task team to create a container registry API standard. Its implementation in Dockstore, and other sites, is a key goal of the standards effort and will allow for federated searches across tool registries that implement the GA4GH API.

## Methods

### Implementation

The Dockstore implementation can be divided into four facets: a tool and workflow registration process aimed at authors, a RESTful web API used to power the site, a web application that uses this API, and, finally, a command line utility that interacts with, and launches, tools and workflows present on Dockstore.

***Tool registration process.*** The Dockstore does not itself act as a Docker image host or provide services to build Docker images automatically from source. These services are already provided reliably and at scale by sites, such as Quay.io and DockerHub. Instead, Dockstore provides a registry to link Docker-based tools hosted on Quay.io or DockerHub with tool metadata described in CWL or WDL and checked into a source control repository at GitHub or Bitbucket. It also acts as a workflow registry for CWL or WDL-based workflow definitions hosted on GitHub or Bitbucket. CWL and WDL provide the emerging standard for describing tools and their parameterizations ( Supplementary File 1) along with overall computational workflows that string together multiple tools. This allows Dockstore to be lightweight and focus on the utility of presenting tools and workflows to the community through a searchable web application.

**Figure 2.**
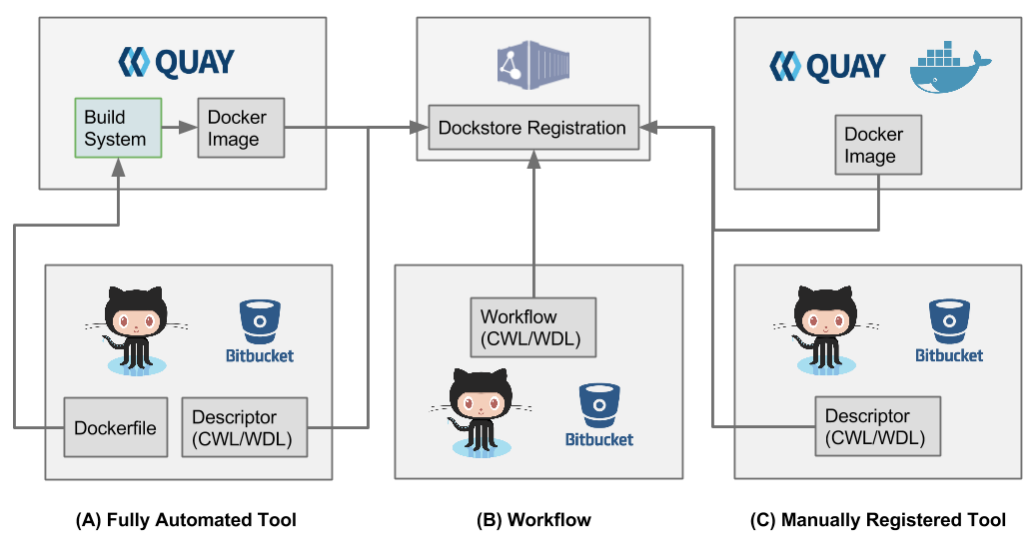
Docker images and tool descriptors or workflows in WDL/CWL are registered with Dockstore. For tools, users can either use the fully automated approach ( **A**) where Docker images are built using Quay.io and original source Descriptors and Dockerfile are on BitBucket or GitHub. Alternatively, they can register pre-build Docker images ( **C**) that have been manually pushed to Quay.io or DockerHub. The former approach results in greater tool transparency and build reproducibility. Workflows in CWL/WDL do not require an image build process and can be directly registered from source control on BitBucket or GitHub ( **B**).

For developers adding tools to Dockstore, we recommend a method in which Docker-based tools are built automatically from public source repositories to maximize transparency and utility to the community. In our preferred approach, Quay.io is used to build the Docker image while GitHub or Bitbucket is used to store the Dockerfile and WDL/CWL descriptor ( Figure 2A). This approach provides a considerable degree of automation for the developer, and encourages practices that result in a clear provenance for the tools during and after development. For example, this approach encourages developers to check in a Dockerfile, the key script used to make reproducible Docker images; the Dockerfile then provides a resource for other users who wish to extend the tool. Multiple releases of a Docker-based tool and its descriptors are supported and clearly associated with each other; the Dockstore web API allows tool developers to register one or more releases of a particular tool with a simple click in the web application. The Dockstore web API gathers descriptors and Dockerfiles via delegated OAuth authorization ^4^. Similarly, the command line tool supports a highly streamlined registration process for Docker images that are built following this automated process. While it is possible to use DockerHub in place of Quay.io, the lack of a public DockerHub API makes integration into Dockstore less streamlined and introduces manual steps.

In addition to the recommended automated build process, Dockstore offers alternative manual processes that give developers greater control over how their tools are registered. For example, Dockstore supports tools built outside of the normal DockerHub/Quay.io automated build process ( Figure 2C). This allows developers to build Docker-based tools themselves, possibly for performance reasons, and then push the finished image to DockerHub or Quay.io for inclusion in Dockstore. The drawback of this for developers is that the series of manual steps cannot necessarily be easily reproduced, while for end users these approaches can obscure how the Docker-based tool image was created. For these reasons we recommend the fully automated approach to developers sharing tools on Dockstore.

***Workflow registration process.*** Workflows are not directly associated with Docker images. Instead, they reference multiple tools (ideally registered using the Dockstore process). For that reason, registering workflows in either CWL or WDL format is simpler, and only requires the workflow document to be checked into source control in BitBucket or GitHub. It can then be found and registered in the Dockstore ( Figure 2B).

**Figure 3.**
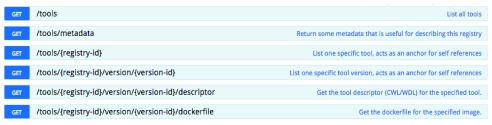
The GA4GH Tool Registry API standard showing the available endpoints. These let systems find all tools in a given repository and get details on a particular tool, including versions, descriptors, and the original Dockerfile if available.

***RESTful application programming interface (API).*** The Dockstore web and command line interfaces are driven by a RESTful web API (DOI: 10.5281/zenodo.154185). This API includes endpoints that conform to the emerging GA4GH Tool Registry API standard ( Figure 3), allowing for multiple tools to interoperate with Dockstore and other sites that implement the standard. The API, currently in its 1.0.0 release, allows for read only access to list and retrieve details of registered Docker images on the site, for more information see https://github.com/ga4gh/tool-registry-schemas. The standard defines the JSON schema used to describe a particular tool registration and includes items such as name, description, author information, tool versions, and test data, in addition to endpoints that allow for listing and filtering tools. In addition, the Dockstore API includes extended, non-standard endpoints that are used for additional features implemented on the site, such as user authentication, integration with third-party services, like Github, and tool labelling.

**Figure 4.**
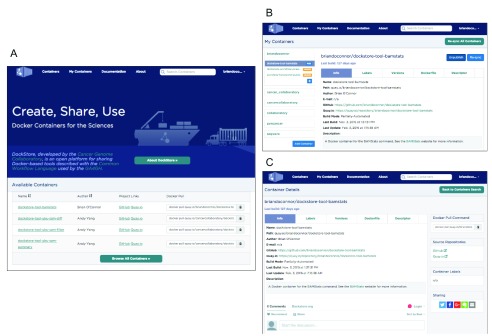
The web interface for the https://dockstore.org site. ( **A**) The main page lists the most recent additions to Dockstore and allows for users to search and login. ( **B**) A developer can easily publish their tools in Dockstore after logging in and linking to accounts. ( **C**) Users can see details about each tool, discuss the tool, share with social media, and navigate back to source.

***Web application interface.*** The Dockstore provides a simple-to-use web application that allows developers to register and manage tools and workflows while enabling end users to find and execute them. The site prominently displays search capabilities on the home page along with recently registered tools ( Figure 4A). The search capability indexes names, descriptions, and versions and presents a list of matching tools. Once a user selects a given tool, the details are displayed, including links out to the Docker hosting service (Quay.io or DockerHub) for tools and the source repository (Bitbucket or GitHub) for tools and workflows ( Figure 4C). The site also includes the ability for authors to tag their registered tools with labels that provide additional searchable annotations ( Figure 4B). Together these features allow a user to quickly search for and identify tools and workflows that are available in Docker and are ready for execution in a variety of environments. The Dockstore web application also provides social features. Each entry incorporates Disqus ( https://disqus.com), a comments system, and links to share entries via various social media sites.

Developers wishing to share their tools on Dockstore can log in using GitHub as an identity provider. Upon first login, they are presented with an onboarding wizard that assists in linking third party services that provide source code hosting (in order to host CWL and WDL descriptors) and Docker registries (in order to host Docker images). For source code, GitHub is linked to by default while Bitbucket is also supported. For Docker images, Quay.io is supported (DockerHub linking is not required since an API is not offered). Once linked, the developer is prompted to download and configure the Dockstore command line tool and is presented with an API token to be used with the underlying Dockstore web service. Developers wishing to build on top of Dockstore can use this token to authenticate against the Dockstore API and use it to make secure requests to GitHub, Bitbucket, and Quay.io.

Following login through GitHub and the onboarding process to set up linked accounts and obtain the command line and API token, the developer is presented with a listing of the Docker images they have previously built with Quay.io. In the recommended build process, we link to the source code repository for the automated build in order to locate tool descriptors. By default the developers’ images are “unpublished” and not publicly visible in Dockstore. Valid images (images that can be linked to a WDL/CWL descriptor) can be toggled to “published”, making them visible to any Dockstore user ( Figure 4B). The developer can use this interface to customize the WDL/CWL paths used, hide or show particular Docker image versions, and add labels to the tool. They can also “refresh” the particular Docker tool, causing Dockstore to re-query Quay.io and GithHub/Bitbucket to ensure the latest build image and associated descriptors are present in the system. For Docker images hosted in DockerHub, a more labor intensive process is needed to manually register in Dockstore given the current lack of publically available DockerHub API. Workflows are registered via a simpler process, since only the path to a CWL or WDL workflow document in GitHub or BitBucket is required.

***Command line interface.*** The Dockstore command line utility provides the registration and search functionality offered by the web interface, and additionally provides assistance for file provisioning and local execution of tools and workflows registered within the system. This functionality allows Dockstore users to find workflows and tools of interest and quickly execute them using a completely standardized approach. Since every tool and workflow in Dockstore is described with CWL or WDL, the local execution of these tools is always done using the same command line and same parameterization process, greatly simplifying the learning curve for using any particular tool or workflow from Dockstore.

Local execution functionality proceeds through three distinct steps: 1) input files are staged; 2) cwl-runner (for CWL descriptors; https://github.com/common-workflow-language/cwltool) or Cromwell (for WDL descriptors; https://github.com/broadinstitute/cromwell) is called to invoke the tool in Docker or the workflow on the local host; and 3) output files are collected and staged to a final location. The parameterization of the Docker-based tool or workflow is encoded in a JSON document, a template of which can be created with the Dockstore command line. The command line launcher supports file downloads from HTTP/HTTPS, Amazon S3, FTP/SFTP, and local file paths, while file uploads are supported for Amazon S3, FTP/SFTP, and local file paths. The Dockstore command line supports file provisioning, since provisioning of files is beyond the scope of the specifications for CWL and WDL. The ability to execute tools from Dockstore is of particular value for development and user evaluation purposes and the command line supports a batch processing mode as well. We anticipate that other systems, both open source and commercial, and through a standard API, will ultimately enable larger-scale concurrent analysis with Dockstore-registered workflows and tools.

### Open source operation

Dockstore follows best practices for software development, including using source control through GitHub, continuous integration testing with Travis CI ( https://travis-ci.org/), testing coverage prediction with Coveralls ( https://coveralls.io/), and community engagement with Gitter ( https://gitter.im/ga4gh/dockstore). The Dockstore web application, https://dockstore.org, will remain an open and free site for users to register their public tool images and workflows. As an open source project ^5^, we also encourage others to customize and install instances of Dockstore (both the UI and RESTful web API) at their own sites. Modifications to the source should be submitted back to the project via the standard GitHub “pull request” mechanism. We hope sites with sharable content participate in our federated network of GA4GH Tool Registry API compliant services, see https://github.com/ga4gh/tool-registry-schemas.

Since Dockstore is designed to use Quay.io and Dockerhub as a backend, the server resources necessary for running it are modest. We recommend a Linux server or VM with 1–4 cores, 8GB of RAM, and 20GB of available disk space. Dockstore has been successfully installed on Ubuntu 14.04 and, while other distributions are possible, we currently only recommend this one.

## Use cases

Dockstore is a general platform for sharing tools and workflows, so the potential use cases the site supports are quite varied. However, we had three primary use cases in mind as the site was built: developers, individual users, and distributed projects performing large-scale computations ( Figure 1).

### Developers

The developer use case focuses on providing a standardized, best-practice development process for building portable tools and workflows. Using Dockstore necessitates that a tool or workflow author uses source control, leverages a Docker build/hosting service, and provides a standardized description of how to invoke the tool/workflow. This development process ensures a given tool or workflow is ready for distribution in a transparent and portable way. Standardized descriptor formats (in WDL or CWL) mean that the tool or workflow is self-documenting, easing the documentation burden on developers. Example Dockerfile, CWL-descriptor, and JSON parameterization files for the BAMStats ( http://bamstats.sourceforge.net) tool can be found in the Supplementary materials ( Supplementary Files 1–3). As an outcome of registering their tools/workflows on Dockstore, developers can take advantage of the underlying GA4GH Tool Registry API standard. This means a growing number of services can find and launch tools from Dockstore, providing additional motivation for developers to redistribute tools and workflows using the site.

### Individual users

For individual users, Dockstore is a catalogue of available tools and workflows that all work in a consistent and reliable way. A user can use Dockstore to find tools and workflows of interest to their research and leverage the standard descriptor format, in either WDL or CWL, to provide clear documentation on how to execute the tool/workflow. Furthermore, the inclusion of known-good test JSON documents on Dockstore provide key examples of inputs and expected outputs, something of importance in the bioinformatics community given the variability in file standards ( Supplementary File 3). In addition to providing clear usage information and example inputs/outputs, individual users can leverage Dockstore-based tools and workflows in a growing collection of execution environments that understand the GA4GH Tool Registry API standard supported by Dockstore. Users will also be able to find and use tools from other sites in a standardized way as more tool and workflow repositories support this API.

### Distributed projects

Large-scale, distributed computational projects are a special form of the developer and user use cases above. Since Dockstore was inspired directly from the lessons learned in the highly-distributed PCAWG project, we feel other large-scale, distributed analysis efforts, such as the upcoming ICGCmed ( https://icgcmed.org) project, will be able to benefit from Dockstore infrastructure. In these projects, Dockstore, or sites supporting the GA4GH Tool Registry standard, provide a standardized way to develop and share portable tools and workflows. Developers and researchers creating analytical tools and workflows for these projects can build, test, and distribute these tools/workflows using Dockstore. This is decoupled from the environments that run the tools and workflows, allowing tool and workflow authors to focus on their scientific content rather than compatibility with execution sites. For those tasked with executing Dockstore-based tools and workflows at scale, their inherent consistency means execution environments shown to run a given Dockstore-based tool or workflow are very likely to be able to run any other Dockstore-based tool or workflow. This separation of concerns, through the consistency provided by Dockstore and portability provided by Docker and standards like CWL and WDL, mean large-scale projects are much more likely to be successful in their distributed computing goals than a model where every tool and workflow needs to be validated across all compute environments used by the distributed project. This is particularly important when environments are changed, added, or removed over the life of the distributed project, or there are a large and dynamic number of tools and workflows being employed, such as in the Dream challenges ( http://dreamchallenges.org/).

## Discussion

The Dockstore is unique in its synthesis of programmatically friendly tool descriptors (WDL or CWL) with Docker images hosted on high-quality commercial services. Together these two features allow tools to be utilized in a variety of automated systems, programmatically discovered, built into larger workflows, and shared with the community. These features are key to supporting the next generation of large-scale genomics analysis projects, such as ICGCmed which require a robust mechanism to encapsulate and move algorithms to data, integrate the efforts of multiple developers, and handle change management in a dynamic environment.

In contrast with generic Docker repositories, such as DockerHub, the Dockstore provides mechanisms to interpret the contents of one or more Docker images, link them together, and execute them on a variety of HPC and cloud environments without modification. Projects like Galaxy Toolshed ^6^ and Bioconda ( https://bioconda.github.io) provide methods for describing and linking tools, but do not use Docker to abstract the execution environments. Hence, the Dockstore approach combines the cloud-based flexibility and elasticity of Docker with the modularity of tool repositories like Galaxy Toolshed.

A number of existing projects, such as BioShaDock ^7^, Bioboxes ^8^, and BioDocker ( http://biodocker.org), focus on encapsulating bioinformatics tools in Docker images in a way similar to Dockstore. BioDocker encourages the use of bioinformatics tools in Docker images by curating them in a single GitHub repository that collaborators can contribute to. Bioboxes defines guidelines ( https://github.com/bioboxes/rfc) for particular types of software, such as assemblers or binning applications, allowing for easy benchmarking and interoperability between tools in bioinformatics pipeline. BioShaDock is the most similar to Dockstore and provides a fully controlled environment to build and publish bioinformatics software. It also hosts Docker images locally. Dockstore, like these existing efforts, encourages the use of Docker as a technology for packaging and distributing bioinformatics tools. However, unlike Bioboxes and BioDocker, Dockstore has a heavy focus on CWL/WDL in order to collect Docker images that can be used as part of larger workflows. Unlike BioShaDock, Dockstore is a lightweight registry that focuses on deep integration with commercial source code providers and the Quay.io Docker image registry. We believe that the combination of a standardized descriptor for bioinformatics tools and integration with third party services allows for a great deal of flexibility by allowing for a robust software development experience, which will enable execution of tools in any CWL/WDL-compatible cloud environment. Furthermore, integration with commercial providers allows for a convenient registration experience that mimics popular services focused on the general software development community, such as Coveralls ( https://coveralls.io/) and Travis CI ( https://travis-ci.org/).

In the future, it should be possible to leverage multiple open source user interfaces (such as Galaxy) and commercial platforms (such as Seven Bridges Genomics, DNAnexus, DNAstack, and others) to provide a friendly environment for finding, combining, and executing Dockstore-based tools and workflows. To further this goal, the creation of the Tool Registry API standard through the GA4GH will be key for future interoperability between tool registries and the systems that scale the execution of tools they contain. The Dockstore is the first implementation of this emerging standard. We hope that other tool repositories will implement the standard, allowing the creation of a tool sharing network of registries. Multiple sites that have different models of how Docker-based tools should be built, shared, and secured, such as BioShaDock, Bioboxes, and BioDocker ( http://biodocker.org) can flourish independently, but benefit from supporting the emerging GA4GH API standard. Such a network stands a good chance of gaining the critical mass to make scientific tool sharing a popular reality

Future features of Dockstore will include the support of testing frameworks and execution environments. The ability to specify test datasets for each tool and workflow will be extended providing users with “known good” sample inputs for testing and instructional purposes. We will also add support for signed Docker images, providing a mechanism to support “verified” Dockstore entries that are validated to come from trusted sources. This will complement private registry support in Dockstore in order to facilitate sharing Docker-based tools and workflows with a select set of collaborators. A long term evolution of the Dockstore site will include a central registry index, complete with faceted search, for querying across the network of GA4GH-compliant tool registries described previously. Dockstore will also integrate with the related and complementary GA4GH Workflow and Task Execution API standards currently in development, enabling the use of compute resources to run Dockstore-based tools and workflows through standardized APIs. Dockstore’s support of these features, and emerging standards, will support future successors to large scale, distributed analysis projects such as PCAWG. This may include efforts, such as the ICGCmed ( https://icgcmed.org/) and future DREAM challenges ( http://dreamchallenges.org/), where Dockstore can enable the seamless interchange and execution of software tools across a variety of computer environments.

## Software availability

Software available at: https://dockstore.org/

Dockstore source code available from the Global Alliance for Genomics and Health (GitHub): https://github.com/ga4gh/dockstore (web UI: https://github.com/ga4gh/dockstore-ui)

Archived source code for Dockstore 1.0 release: https://zenodo.org/record/154185, DOI: 10.5281/zenodo.154185 ^9^

License: Apache 2.0

## Supplementary material

Supplementary File 1: Zipped file containing the following (

.):

A tool descriptor, in this case the **Dockstore.cwl** descriptor written for the BAMStats tool on Dockstore ( https://dockstore.org/containers/quay.io/collaboratory/dockstore-tool-bamstats). Descriptors define the key attributes like name, inputs and outputs of a tool, the system requirements, which Docker image to use, authorship information, and information making the construction of the command possible.

A **Dockerfile** that includes the instructions on how to make a Docker image, in this case, one containing the BAMStat tool.

This **Sample.json** file provides sample parameterizations for this tool including a “known good” input BAM file.
