# Supplementary material for: The Dockstore: enabling modular, community-focused sharing of Docker-based genomics tools and workflows
Source: F1000Res. 2017 Jan 18;6:52. [Version 1] doi: 10.12688/f1000research.10137.1 (PMC5333608; doi:10.12688/f1000research.10137.1)
Supplement: Peer review history

# Peer review: Referee response for version 1

**DOI:** 10.5256/f1000research.10919.r20278

**Published:** 2017-02-27

**Authors:** Heinz Stockinger

**Affiliations:** 1 SIB Swiss Institute of Bioinformatics, Lausanne, Switzerland

The article is very well written and discusses the implementation of a valuable tool for the community. The use of Docker is currently very popular, and the combination with CWL/WDL is very good.

 I have a minor comment for the on-line tool and the presented workflows (https://dockstore.org/search-workflows). Currently, there does not seem to be description for the presented workflows. Example:

 https://dockstore.org/workflows/ICGC-TCGA-PanCancer/wdl-pcawg-bwa-mem-workflow

 "No description associated with this workflow. "

 This makes it difficult for users to select a workflow. Adding a short description would be very helpful.

I have read this submission. I believe that I have an appropriate level of expertise to confirm that it is of an acceptable scientific standard.

# Peer review: Referee response for version 1

**DOI:** 10.5256/f1000research.10919.r19471

**Published:** 2017-02-01

**Authors:** Gaurav Kaushik

**Affiliations:** 1 Seven Bridges Genomics, Inc., Cambridge, MA, USA

In this manuscript, the authors describe the motivation, design, architecture, and merit of Dockstore.org, a community-focused utility for sharing Docker-based tools and workflows for the sciences.

 The authors should be commended for their overview of the significant challenges facing large-scale genomics efforts, such as maintaining consistent, reproducible analyses across environments, as well as the solution they’ve architected. They highlight important considerations that must be addressed in order to accelerate scientific progress and the improvement of human health. The technical description of the ICGC PCAWG project is illuminating for researchers and organizations wanting to organize or participate in large-magnitude informatics projects.

 Overall, we recommend that the manuscript be accepted pending minor revisions. Each revision item is discussed below:

 The description of Dockstore architecture is thorough and each design decision is justified and informative to the reader. A few additions, however, may benefit audiences which are less conversant in Docker or cloud architecture. For example, though container-based workflow descriptions are becoming increasingly common, many researchers may not yet be familiar with CWL and WDL. A more detailed description of the container-tool-workflow relationship and the benefit of modularizing workflows into containerized tools (as opposed to have whole workflows in a single container) may be helpful to newcomers.

 We request that the authors cite the Common Workflow Language and Workflow Description Language as appropriate. For CWL, the appropriate citation is https://dx.doi.org/10.6084/m9.figshare.3115156.v2, as stated on commonwl.org. ^1^ For WDL, we have previously cited their GitHub repository ( https://github.com/broadinstitute/wdl) though a more appropriate citation may now exist and could be provided by their development team.

 The authors mention that cloud APIs and scripts resulted in analytical failures. The manuscript may benefit from brief discussion of any design constraints when using containers and workflows that may introduce similar risks. If there are none or relatively few, please elucidate why such a technological advantage exists to the reader.

 Figure 2 may benefit from streamlining, as there are duplicate images and the discussion items (A-C) are mentioned out of order.

 Regarding the use of GitHub for automated builds and workflow descriptions, the reader may benefit from a small description of best practices in a supplement. For example, how does Dockstore handle tagging of Dockerfiles and how should users make use of them? This can be brief, but it may be helpful to better describe how to augment the value add that Dockstore brings to reproducibility with good git practices.

 On page 7, “Dream” should be “DREAM”.

I have read this submission. I believe that I have an appropriate level of expertise to confirm that it is of an acceptable scientific standard.

## References

[1] AmstutzPCrusoeMRTijanićNChapmanBChiltonJHeuerMKartashovALeehrDMénagerHNedeljkovichMScalesMSoiland-ReyesAStojanovicL: Common Workflow Language, v1.0.2016; 10.6084/m9.figshare.3115156.v2 10.6084/m9.figshare.3115156.v2
